# A subset of gastric cancers with EGFR amplification and overexpression respond to cetuximab therapy

**DOI:** 10.1038/srep02992

**Published:** 2013-10-21

**Authors:** Lianhai Zhang, Jie Yang, Jie Cai, Xiaoming Song, Jianyun Deng, Xuesong Huang, Dawei Chen, Mengmeng Yang, Jean-Pierre Wery, Shuangxi Li, Aiwen Wu, Ziyu Li, Zhongwu Li, Yiqiang Liu, Yiyou Chen, Qixiang Li, Jiafu Ji

**Affiliations:** 1 Key laboratory of Carcinogenesis and Translational Research (Ministry of Education), Department of Surgery; 2 Department of Pathology, Peking University Cancer Hospital and Institute, Beijing, China; 3 Crown Bioscience Inc. (Beijing), Light Muller Building, ChangPing Sector of Zhongguanchun Science Park, No. 21 Huo Ju St., ChangPing District, Beijing, China; 4 State Key Laboratory of Natural and Biomimetic Drugs, Peking University, Beijing, China; 5 These authors contributed equally to this work.

## Abstract

A preclinical trial identified 4 of 20 (20%) gastric cancer (GC) patient-derived xenografts responded to cetuximab. Genome-wide profiling and additional investigations revealed that high EGFR mRNA expression and immunohistochemistry score (3+) are associated with tumor growth inhibition. Furthermore, EGFR amplification were observed in 2/4 (50%) responders with average copy number 5.8 and >15 respectively. Our data suggest that a GC subtype with EGFR amplification and overexpression benefit from cetuximab treatment.

Gastric cancer (GC) is one of the leading causes of cancer mortality worldwide. The treatment outcome is poor for majority of GC patients^1^. Modest efficacy and considerable toxicities associated with chemotherapy have prompted the pursuit of novel therapy targeting genetic and molecular alterations that drive gastric carcinogenesis. Trastuzumab is the only approved target agent for a subgroup of GC patients with HER2 overexpression at present, which represent about 20% of all the patients^2^, based on the results of phase III ToGA trial^3^. There is an urgent need for more effective target agents for treating this disease.

Cetuximab is a recombinant human/mouse chimeric monoclonal antibody against EGFR. Cetuximab was approved for treating EGFR-expressing metastatic CRC (mCRC) without activating KRAS mutation, and squamous cell carcinoma of the head and neck (SCCHN)^4^, but yet for GC. Several phase II trials have evaluated cetuximab as a first-line treatment in combination with various chemotherapy regimens^5^,^6^,^7^,^8^, demonstrating response in a subset of GC patients with overall response rate (ORR) of 40–60%. However, a randomized phase III trial, EXPAND (Erbitux in Combination With Xeloda and Cisplatin in Advanced Esophago-gastric Cancer, NCT00678535) did not significantly increase progression-free survival (PFS) in patients with advanced GC^9^. Unlike HER2 in GC, the predictive value of increased EGFR copy number for tumor response and skin rash are controversial^6^,^8^. At present, there is no established biomarker to predict response to cetuximab.

There has been an increase in using experimental models to predict clinical activity of agents and discover predictive biomarkers. Patient-derived tumor xenografts (PDXs), also called as “avatar mice” or “xenopatients”, mirror patients' histopathological and genetic profiles^10^,^11^,^12^,^13^,^14^. Large collection of them reflects diversity of tumors in patient populations. We have established a large collection of cancer PDXs by transplanting surgically removed tumor tissues from patients into immunocompromised BALB/c nude mice via subcutaneous inoculation, including many gastric cancer PDXs (GC-PDXs), to assess drug activities^15^.

This study investigated the activity of cetuximab in 20 GC-PDX models. After therapeutic responders and non-responders were identified, following discovery of predictive biomarkers including genomic and gene expression analysis, sequence of key oncogenes was carried out. And the expressions of candidate biomarkers were validated by quantitative PCR, immunohistochemistry, and fluorescence in-situ hybridization (FISH).

## Results

### A subset of GC xenografts responded to cetuximab

We established GC-PDX models by transplanting surgically removed tumor tissues from GC patients into immunocompromised Balb/c nude mice via subcutaneous inoculation. Then we set out to test a cohort of randomly selected 20 GC-PDXs in a clinical trial-like study to assess cetuximab activities by subjecting them to the drug treatment (50 mg/kg, intraperitoneally, IP) once weekly for 2 weeks. The original patient clinicopathological features, along with the model pathology confirmation, are summarized in Supplementary Table 1.

**Figure 1:**
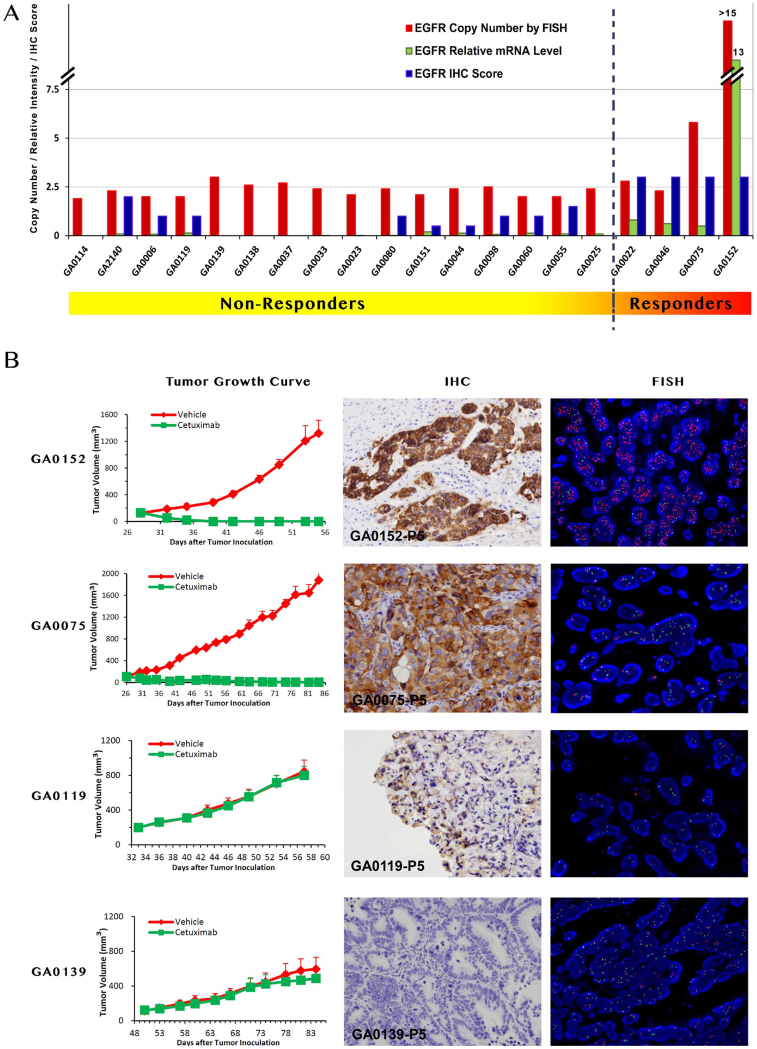
The response to cetuximab treatment and genetic profile of GC-PDX models. Panel A: The PDX-GC models are sorted by the tumor response to cetuximab (ΔT/ΔC). The responders at the right part display higher EGFR mRNA level and IHC staining intensity, and the only two cases (GA0075 and GA0152, CN > 5) of gene amplification. Panel B: The representative images of responders and non-responders. The responders GA0152 and GA0075 display IHC score 3+, and gene amplification (GA0075, CN = 5.8; GA0152, CN > 15), while non-responders GA0119 and GA0139 are with IHC low expression and no gene amplification. Left: Representative tumor growth curves of responders and non-responders. Middle: IHC analysis of tumor models; Right: Dual-color FISH assay in gastric carcinoma. Probe for EGFR locus is labeled in red and CEP7 labeled in green. Blue: Nuclei.

The tumor response to cetuximab is quantified by ^ΔT^/_ΔC_^15^ and summarized in Table 1. The tested GC-PDXs fall into two distinct categories according to the drug activities: 4 of 20 (20%) responded with nearly complete response (^ΔT^/_ΔC_ < 0) to cetuximab treatment; 16 of 20 (80%) did not, with partial or complete resistance (ΔT/ΔC > 30%). The representative tumor response curves are shown in the left column of Figure 1B. GA0152 and GA0075 are examples of cetuximab sensitive models, while GA0119 and GA0139 are resistant models. Our data clearly suggest that a subset of GC tumors can potentially benefit from cetuximab treatment.

### About 50% responders display EGFR gene amplification

In order to discover potential predicting markers of cetuximab response, therefore, we performed molecular characterization of these models, including genome-wide copy number variation and transcriptome profiling, First, we interrogated copy number variation of GC-PDXs using Affymetrix genome-wide human SNP6.0 array and PICNIC (Predicting Integral Copy Numbers In Cancer) algorithm^15^. We found that EGFR copy numbers of all four responders are higher than most of those non-responders (Table 1, *P* = 0.002). To further confirm this finding, we assessed EGFR gene copy number by real-time quantitative PCR (q-PCR) and found that all responders have copy number ≥4, while only 2 of 16 (12.5%) non-responders have copy number ≥4. The difference between these two group is significant (*P* = 0.008). The highest value, 15 by SNP6 + PICNIC analysis and 1040.9 by q-PCR, is from GA0152, which is also the best responder.

To further confirm the EGFR gene amplification, we further performed fluorescence in situ hybridization (FISH), a more accurate assay used to determine HER2 gene amplification for guiding anti-HER2 treatment for advanced GC in the clinical practice. At least 100 non-overlapping interphase nuclei were observed for the number of copies of EGFR. EGFR status was scored as the number of EGFR signals per nucleus. Our result demonstrated EGFR amplification in 2/4 (50%) responders with average copy number 5.8 (GA0075) and >15 (GA0152), respectively (Fig. 1A, Table 1). GA0152 was also with EGFR/CEP7 ratio >15. Thus, 2/4 (50%) responders could be predicted by EGFR amplification.

### All responders display higher EGFR mRNA expression level

On the other hand, transcriptome profiling using Affymetrix HG-U219 GeneChip, revealed that all of the four responders expressed higher levels of EGFR mRNA expression than all 16 non-responders did (*P* = 0.003) (Table 1). EGFR gene expression was further quantified by q-RT-PCR (quantitative reverse transcription-PCR) against house-keeping gene GAPDH. Among the samples tested, 4 samples exhibited high EGFR mRNA levels (relative intensity ≥0.5, arbitrarily defined) were all responders, in contrast to the remaining models showing medium to low EGFR mRNA levels (relative intensity ≤ 0.1) (Table 1, Fig. 1A). The difference is significant (*P* = 0.002). In particular, the highest value is from GA0152, with 10.5 by GeneChip analysis and 13 by q-RT-PCR, which can be attributed to the EGFR amplification mentioned above.

### All responders display higher EGFR immunohistochemistry score

Then we performed EGFR immunohistochemistry (IHC), a clinically practical assay to determine HER2 expression for anti-HER2 treatment for GC. IHC demonstrated positive EGFR immunostaining in 12/20 (60%) models. Among them, 6/12 had staining intensity score of 1+, 3/12 of 2+, and 3/12 of 3+. All responders were found EGFR IHC 3+, while the non-responders displayed lower EGFR IHC score 0–2+ (*P* = 0.002) (Table 1, Fig. 1A). The typical EGFR strong immunostaining (GA0152 and GA0075) is showed in Figure 1B. These results demonstrated that the EGFR high expression (in both mRNA and protein level) is correlated to the response to cetuximab.

### Mutation of associated oncogenes is rare

Genetic mutations of some common oncogenes associated with EGFR pathway^15^,^16^,^17^,^18^,^19^, e.g. KRAS, BRAF (V600E), c-MET, EGFR, AKT and PI3KC have also been investigated in these models by hot-spot mutation sequencing^15^. Interestingly, few of the tested models, regardless responders or non-responders, showed any aberrations with exception of GA0139 containing G13D KRAS mutation, GA0044 containing 327–329 deletion in PIK3CA, and GA0098 containing G545Y PIK3CA mutation (Table 1). Therefore, the non-response of GC xenografts to cetuximab apparently cannot be simply attributed to these oncogene mutations.

## Discussion

Our data point to a positive correlation between cetuximab response in GC and the EGFR high expression at both mRNA and protein level, as well as EGFR gene amplification. This correlation is exemplified by GA0152 that has the highest EGFR mRNA expression, IHC score and gene amplification. The data seem to suggest the higher activity of EGFR via higher expression drives the oncogenic transformation in these tumors, and therefore its inactivation by cetuximab thus inhibits tumor growth. Overexpression of EGFR could be attributes to the gene amplification in two cases, however, the exact mechanism of EGFR high expression in the other two cases has yet to be investigated.

A recent phase II trial^8^, with cetuximab combined therapy for GC (European Clinical Trials Database number 2004-004024-12) showed association between higher EGFR copy number (defined as ≥4, 8 of 36 cases, 22.2%; including 1 amplification case ≥6 and FISH positive) and better overall survival^20^. Their clinical data seem to be consistent with our data in this mouse clinical trial that all responders display higher EGFR gene copy number ≥4 while only two (50%) are FISH positive. Our data also demonstrated the EGFR high expression in both mRNA and protein level is correlated to the response. However, since the mRNA expression of EGFR genes is not routinely assayed in the clinical samples, and IHC can be of controversy due to biological and technical factors, we recommend that the combination of FISH and IHC tests are suitable for predicting cetuximab efficacy as routine clinical practice, similar to the clinical practice of anti-HER2 treatment.

In summary, our study suggests that a GC subtype with high EGFR mRNA expression and IHC score 3+ may benefit from cetuximab treatment, and the EGFR gene amplification by FISH can also accurately predict the responders with positive predictive value around 50%. These markers can be helpful for guiding future a potentially successful clinical trial and eventually as a patient stratification guide for clinical treatment.

## Methods

### Patient tumor samples and engraftment in immunocompromised mice

Freshly and surgically removed tumor tissues were obtained from the patients diagnosed as GC in Peking University Cancer Hospital through approval by the Institutional Review Boards of the hospital and the informed consents from all patients. The engraftment of patient tumor fragments into immunocompromised mice subcutaneously was previously described^15^. Briefly, the tumors were sliced into 3 × 3 × 3 mm^3^ fragments and inoculated subcutaneously on the flank of mice (BALB/c nude, 6- to 8-weeks old female mice, Beijing HFK Bioscience Co., Beijing, China). The tumor growth was monitored twice weekly using a caliper. The established tumor models, called passage 0 or P0, were serially re-engrafted to maintain tumors in vivo. These subsequent passages were called P1, 2, 3… (<10). When tumors sizes reach 500–700 mm^3^ (1/2 length × width^2^), they were harvested for the next round of engraftment for serial passage or conducting studies of pharmacology, histopathology, immunohistology, cellular and molecular analysis. All procedures were under sterile conditions at Crown Bioscience SPF facility and conducted in strict accordance with the Guide for the Care and Use of Laboratory Animals of the National Institutes of Health. The protocol was approved by the Committee on the Ethics of Animal Experiments of Crown Bioscience (Crown Bioscience IACUC Committee).

### Evaluation of antitumor activity

When tumor volume reaches 100–150 mm^3^, the mice were randomly grouped into two groups of five mice with similar average tumor volume. Immediately after grouping, the control group was treated with vehicle (PBS, weekly intraperitoneal injection or IP for 2 weeks), and the treatment groups were injected with cetuximab (weekly IP injection for 2 weeks, 50 mg/kg, Merck KGaA). The tumor growth was monitored twice weekly, and ^ΔT^/_ΔC_ value was calculated for assessing tumor response to the treatment (ΔT = tumor volume change in the treatment group and ΔC = tumor volume change in the control group). The total number of the mice for xenograft is 200 (10 mice/model for 20 PDX models).

### EGFR IHC analysis of GC tumors

Standard immunohistochemistry (IHC) was used to analyze tumor tissues from the PDX xenograft models. Briefly, the tissues were fixed in 10% neutral buffered formalin and embedded in paraffin per standard histological procedures. After deparaffinization and rehydratation, 3-μm thick tissue sections were pretreated in 0.01 M sodium citrate, pH 6.0 solution at 95°C for 30 min, followed by staining with rabbit anti-human EGFR antibody (Cell Signaling, Boston, USA) at final dilution 1:200. Positive staining was detected using Detection System HRP Polymer Kit (Lab Vision, Fremont, USA). DAB was used as the chromogenic substrate, and sections were counterstained with Gill's hematoxylin (Fisher Scientific, Fair Lawn, NJ). The test specimens were then scored independently by three investigators in a blinded fashion per following criteria recommended by Shia et al in 2005^21^: Score 0 is when there was no specific membrane staining within the tumor, and positive when there was any staining of tumor cell membrane above background level. The positive cases were further classified into 1+, 2+ and 3+ based on the staining intensity of the membrane.

Areas of most intensity were identified by scanning tumor sections at low power (100×), and then images were photographed at high magnification (400×) using Olympus BX51 microscopy system with DP71 digital camera (Olympus, Melville, NY).

### Gene expression profiling and gene copy number analysis of GC-PDX

Fresh GC-PDX tumor tissues were collected from the tumor-bearing mice, snap-frozen and stored at −80°C before being used for genetic and genomic analysis. For gene profiling analysis, the total RNA was isolated from the frozen tissues using Trizol (Invitrogen, Carlsbad, CA) per the manufacturer's instructions, and purified using RNeasy mini columns (Qiagen). RNA quality was assessed on a Bioanalyzer (Agilent). Only RNA samples with high quality (RIN > 8) were used for expression profiling assays on Affymetrix HG-U219 array plates following standard protocol (http://media.affymetrix.com/support/downloads/manuals/3_ivt_express_kit_manual.pdf). Raw CEL data sets of all samples were normalized by RMA algorithm. Probe set intensity was expressed as log(2) transformed values. For CNV assay using Affymetrix SNP6.0 chips, genomic DNA was isolated and purified using Genomic DNA Tissue and Blood Isolation Kit (Qiagen) following manufacturer's instruction. DNA processing and chip hybridization were performed following standard Affymetrix protocol (http://media.affymetrix.com/support/downloads/manuals/genomewidesnp6_manual.pdf). Raw CEL data were QC-ed and filtered to remove low call-rate samples, and gene copy number analysis were performed by PICNIC and/or PennCNV methods.

For all of the samples, the relative EGFR gene expression level was determined by quantitative RT-PCR. Extracted mRNA was subjected to amplification using human EGFR specific primers by TaqMan q-PCR. The human GAPDH gene was used as a reference. TaqMan probes and primers for EGFR (assay ID: Hs01076078_m1), GAPDH (Assay ID: Hs99999905_m1) were obtained from Applied Biosystems. The raw data generated by the system were processed using the ΔCT relative quantification. ΔCT = (CT value of target gene) - (CT value of reference gene). ΔCT values were then converted into intensity value (relative mRNA level = 2^∧^ (−ΔCT).

Also, EGFR gene copy numbers were determined by quantitative PCR. Briefly, the same genomic DNAs were subjected to amplification by TaqMan q-PCR. The primers for EGFR (assay ID: Hs04960197_cn) and RNase P as endogenous reference (part number 4401631) were purchased from Applied Biosystems. The raw data was transferred to CopyCaller software and analyzed.

### FISH

Three micrometer thick tissue sections were treated with the procedure provided by fluorescence in-situ hybridization (FISH) detection kit (DakoCytomation, Glostrup, Denmark). Samples were placed in pretreatment solution for 30 min at 96°C, and digested with pepsin solution for 30 min at room temperature. Dual-color, dual-target FISH assays were done with the EGFR Spectrum Orange/CEP7 Spectrum Green Probe (Vysis, USA). Tissue sections, covered with 10-μL probe solution, were incubated at 75°C for 5 min to co-denature the EGFR and CEP7 (chromosome seven α-centromeric) probes and allowed to hybridize overnight at 37°C. Co-denaturation and hybridization were done sequentially. Post-hybridization stringency wash was done in a water bath at 65°C for 10 min. After washing twice and drying at room temperature for 15 min, tissue sections were covered with 4′6-diamidino-2-phenylindole (DAPI II, Vysis, USA) for chromatin counterstaining.

Analyses were done with a fluorescence microscope (Zeiss Axiophot, Germany) equipped with a Metachrome II cooled-charged device camera (Zeiss, Germany). EGFR was visualized as a red signal with a standard TRITC (tetramethyl rhodamine isothiocyanate) filter, CEP7 as a green signal with a FITC (fluorescein isothiocyanate) filter, and nuclei as a blue signal with a DAPI filter. Representative images of samples were acquired and then analyzed.

Two independent observers scored at least 100 non-overlapping interphase nuclei for the number of copies of EGFR and CEP7 by use of predefined scoring guidelines. EGFR status was scored as the number of EGFR signals per nucleus and as the ratio of EGFR signals to CEP7 signals. Negative controls consisted of a cultured retinal pigment epithelial (RPE) cell line; the control for amplified EGFR was the A431 cell line derived from human epidermoid carcinoma. Amplification was defined as the presence of 5 or more signals per nucleus, i.e., EGFR copy number ≥5.

### EGFR mutation analysis

Gene hotspot analyses of common oncogenes associated with resistance to cetuximab such as EGFR (Exon18;19;20;21), KRAS (Exon2;3;4), BRAF (Exon15;V600E), c-MET (Exon14;16;17;18;19;21), PI3KC (Exon1;9;20) were carried out to identify the mutations in the tumors. Briefly, genomic DNA was extracted from the tissues using kit mentioned above according to the manufacturer's instructions. Primers used for mutation analyses are shown in Supplementary Table 2.

Polymerase chain reaction was performed in 50 μL reaction mixtures containing: 100 ng of genomic DNA, 5 μL 10× PCR buffer, 0.2 μM each of primers, 0.2 mM 4× dNTPs and 1 μL TaqE. Reaction was carried out for 40 amplification cycles. The amplified PCR products were gel purified and sequenced by Sanger Automated Sequencer (ABI). The specificity of the primers to human genes had been assured by BLAST search. Sequencing data alignment analysis and mutation identification was performed using BioEdit software.

### Statistical analysis

The nonparametric Mann-Whitney U test was applied for comparing of the profiling data of two groups, i.e., non-responders and responders. In all analyses, p < 0.05 was considered statistically significant. The statistical analysis was carried out with SPSS V13.0 software (SPSS Inc., Chicago, IL, USA).

## Author Contributions

Q.L. and J.J. conceived and designed the experiments; L.Z., J.Y., J.C., X.S., J.D., X.H., D.C., M.Y., J.P.W., S.L., A.W., Z.L., Z.L., Y.L. and Y.C. performed the animal model construction and experiments; L.Z. and J.Y. analyzed the data and contributed to writing and editing the manuscript; Q.L. and J.J. supervised the project and wrote the manuscript.

## Supplementary Material

Supplementary Information Supplementary Tables

## Figures and Tables

**Table 1:** Treatment response, EGFR status and mutation status of GC PDX models

	Non-Responders																Responders				
	GA	GA	GA	GA	GA	GA	GA	GA	GA	GA	GA	GA	GA	GA	GA	GA	GA	GA	GA	GA	*P* value (non- vs. responders)
Model ID	0114	2140	0006	0119	0139	0138	0037	0033	0023	0080	0151	0044	0098	0060	0055	0025	0022	0046	0075	0152	
ΔT/ΔC	1.744	1.492	1.42	0.934	0.912	0.898	0.881	0.811	0.781	0.748	0.717	0.687	0.58	0.488	0.41	0.305	−0.071	−0.078	−0.098	−0.121	0.002
Copy Number																					
EGFR (SNP6 + PICNIC)	6	NE*	7	5	5	5	5	5	4	6	NE	5	5	5	5	NE	7	7	8	15	0.002
EGFR (q-PCR)	5.1	3.8	2.1	2.1	4.8	2.7	3.7	3.9	1.9	3.7	1.4	2.1	3.7	3.1	2.9	2.1	5.4	4.3	4.6	1040.9	0.008
EGFR (FISH)	1.9	2.3	2.0	2.0	3.0	2.6	2.7	2.4	2.1	2.4	2.1	2.4	2.5	2.0	2.0	2.4	2.8	2.3	5.8	>15	0.029
CEP7 (FISH)	1.9	2.2	2.1	2.0	2.7	2.0	2.3	2.0	2.5	2.1	2.2	2.3	2.1	2.2	2.0	2.3	2.0	2.3	5.2		
Ratio (EGFR/CEP7)	0.96	1.04	0.97	1.03	1.09	1.29	1.16	1.21	0.83	1.16	0.93	1.03	1.2	0.91	1.01	1.05	1.39	1.03	1.12	>15	0.099
mRNA																					
EGFR U219 intensity	2.9	NE	3.3	3.6	2.9	2.3	2.5	2.4	2.5	2.8	3.6	3.1	4.3	4.2	4	3.8	6.5	6.9	5.8	10.5	0.003
EGFR Relative Intensity	0	0.1	0.08	0.14	0	0	0	0.02	0.02	0.02	0.2	0.13	0.07	0.13	0.1	0.1	0.81	0.62	0.5	13	0.002
Protein																					
EGFR IHC Score	0	2	1	1	0	0	0	0	0	1	1	1	1	1	2	0	3	3	3	3	0.002
Mutation																					
EGFR|Exon18;19;20;21	WT	NE	WT	WT	WT	WT	WT	WT	WT	WT	WT	WT	WT	WT	WT	WT	WT	WT	WT	WT	
k-RAS|Exon2;3;4	WT	NE	WT	WT	G13D	WT	WT	WT	WT	WT	WT	WT	WT	WT	WT	WT	WT	WT	WT	WT	
BRAF|Exon15	WT	NE	WT	WT	WT	WT	WT	WT	WT	WT	WT	WT	WT	WT	WT	WT	WT	WT	WT	WT	
c-MET|Exon14;16;17;18;19;21	WT	NE	WT	WT	WT	WT	WT	WT	WT	WT	WT	WT	WT	WT	WT	WT	WT	WT	WT	WT	
PIK3CA|Exon1;9;20	WT	NE	WT	WT	WT	WT	WT	WT	WT	WT	WT	Del* 327–329	G545Y	WT	WT	WT	WT	WT	WT	WT	

These model are sorted by the ratio of ΔT/ΔC, and the final four are responders.* Del: deletion; NE: not evaluable.

## References

[b1] Jemal A. *et al.* Global cancer statistics. CA. Cancer J. Clin. 61, 69–90 (2011). 21296855 10.3322/caac.20107

[b2] Rose J. S. & Bekaii-Saab T. S. New developments in the treatment of metastatic gastric cancer: focus on trastuzumab. Onco Targets Ther 4, 21–26 (2011). 21552412 10.2147/OTT.S10188 PMC3084304

[b3] Bang Y. J. *et al.* Trastuzumab in combination with chemotherapy versus chemotherapy alone for treatment of HER2-positive advanced gastric or gastro-oesophageal junction cancer (ToGA): a phase 3, open-label, randomised controlled trial. Lancet 376, 687–697 (2010). 20728210 10.1016/S0140-6736(10)61121-X

[b4] Bonner J. A. *et al.* Radiotherapy plus cetuximab for locoregionally advanced head and neck cancer: 5-year survival data from a phase 3 randomised trial, and relation between cetuximab-induced rash and survival. Lancet Oncol. 11, 21–28 (2010). 19897418 10.1016/S1470-2045(09)70311-0

[b5] Pinto C. *et al.* Phase II study of cetuximab in combination with FOLFIRI in patients with untreated advanced gastric or gastroesophageal junction adenocarcinoma (FOLCETUX study). Ann. Oncol. 18, 510–517 (2007). 17164226 10.1093/annonc/mdl459

[b6] Han S. W. *et al.* Phase II study and biomarker analysis of cetuximab combined with modified FOLFOX6 in advanced gastric cancer. Br. J. Cancer 100, 298–304 (2009). 19127259 10.1038/sj.bjc.6604861 PMC2634707

[b7] Kim C. *et al.* A prospective phase II study of cetuximab in combination with XELOX (capecitabine and oxaliplatin) in patients with metastatic and/or recurrent advanced gastric cancer. Invest. New Drugs 29, 366–373 (2011). 19997960 10.1007/s10637-009-9363-0

[b8] Lordick F. *et al.* Cetuximab plus oxaliplatin/leucovorin/5-fluorouracil in first-line metastatic gastric cancer: a phase II study of the Arbeitsgemeinschaft Internistische Onkologie (AIO). Br. J. Cancer 102, 500–505 (2010). 20068568 10.1038/sj.bjc.6605521 PMC2822949

[b9] Lordick F. *et al.* Capecitabine and cisplatin with or without cetuximab for patients with previously untreated advanced gastric cancer (EXPAND): a randomised, open-label phase 3 trial. Lancet Oncol. 14, 490–499 (2013). 23594786 10.1016/S1470-2045(13)70102-5

[b10] Marangoni E. *et al.* A new model of patient tumor-derived breast cancer xenografts for preclinical assays. Clin. Cancer Res. 13, 3989–3998 (2007). 17606733 10.1158/1078-0432.CCR-07-0078

[b11] Nemati F. *et al.* Establishment and characterization of a panel of human uveal melanoma xenografts derived from primary and/or metastatic tumors. Clin. Cancer Res. 16, 2352–2362 (2010). 20371695 10.1158/1078-0432.CCR-09-3066

[b12] Nemati F. *et al.* Clinical relevance of human cancer xenografts as a tool for preclinical assessment: example of in-vivo evaluation of topotecan-based chemotherapy in a panel of human small-cell lung cancer xenografts. Anticancer. Drugs 21, 25–32 (2010). 19823076 10.1097/CAD.0b013e3283300a29

[b13] Fichtner I. *et al.* Establishment of patient-derived non-small cell lung cancer xenografts as models for the identification of predictive biomarkers. Clin. Cancer Res. 14, 6456–6468 (2008). 18927285 10.1158/1078-0432.CCR-08-0138

[b14] Hennessey P. T. *et al.* Promoter methylation in head and neck squamous cell carcinoma cell lines is significantly different than methylation in primary tumors and xenografts. PLoS One 6, e20584 (2011). 21637785 10.1371/journal.pone.0020584 PMC3102742

[b15] Yang M. *et al.* Overcoming erlotinib resistance with tailored treatment regimen in patient-derived xenografts from naive Asian NSCLC patients. Int. J. Cancer 132, E74–84 (2013). 22948846 10.1002/ijc.27813

[b16] Lievre A. *et al.* KRAS mutation status is predictive of response to cetuximab therapy in colorectal cancer. Cancer Res. 66, 3992–3995 (2006). 16618717 10.1158/0008-5472.CAN-06-0191

[b17] De Roock W. *et al.* Effects of KRAS, BRAF, NRAS, and PIK3CA mutations on the efficacy of cetuximab plus chemotherapy in chemotherapy-refractory metastatic colorectal cancer: a retrospective consortium analysis. Lancet Oncol. 11, 753–762 (2010). 20619739 10.1016/S1470-2045(10)70130-3

[b18] De Roock W., De Vriendt V., Normanno N., Ciardiello F. & Tejpar S. KRAS, BRAF, PIK3CA, and PTEN mutations: implications for targeted therapies in metastatic colorectal cancer. Lancet Oncol. 12, 594–603 (2011). 21163703 10.1016/S1470-2045(10)70209-6

[b19] Di Nicolantonio F. *et al.* Wild-type BRAF is required for response to panitumumab or cetuximab in metastatic colorectal cancer. J. Clin. Oncol. 26, 5705–5712 (2008). 19001320 10.1200/JCO.2008.18.0786

[b20] Luber B. *et al.* Biomarker analysis of cetuximab plus oxaliplatin/leucovorin/5-fluorouracil in first-line metastatic gastric and oesophago-gastric junction cancer: results from a phase II trial of the Arbeitsgemeinschaft Internistische Onkologie (AIO). BMC Cancer 11, 509 (2011). 22152101 10.1186/1471-2407-11-509 PMC3252322

[b21] Shia J. *et al.* Epidermal growth factor receptor expression and gene amplification in colorectal carcinoma: an immunohistochemical and chromogenic in situ hybridization study. Mod. Pathol. 18, 1350–1356 (2005). 15832190 10.1038/modpathol.3800417

