# Supplementary material for: A subset of gastric cancers with EGFR amplification and overexpression respond to cetuximab therapy
Source: Sci Rep. 2013 Oct 21;3:2992. doi: 10.1038/srep02992 (PMC3801116; doi:10.1038/srep02992)
Supplement: Supplementary Information — Supplementary Tables [file srep02992-s1.pdf]

A subset of gastric cancers with EGFR amplification and overexpression respond to cetuximab therapy

Authors

Lianhai Zhang,[1,5] Jie Yang,[3,5] Jie Cai,[3] Xiaoming Song,[3] Jianyun Deng,[3] Xuesong Huang,[3] Dawei Chen,[3] Mengmeng Yang,[3] Jean-Pierre Wery,[3] Shuangxi Li,[1] Aiwen Wu,[1] Ziyu Li,[1] Zhongwu Li,[2] Yiqiang Liu,[2] Yiyou Chen,[3] Qixiang Li,[3,4*] and Jiafu Ji[1*]

Supplement:

Supplementary Table 1. Clinicopathological parameters of GC patients and the pathology confirmation of corresponding PDX models.

ID	Sex	Tumor Size (cm)	Depth of Wall Invasion	Lymph Node Metastasis	Distant Metastasis	TNM stages	Histology (patients and PDX)	Differentiation
**GA0006**	M	>5.0	T4a	N1	M0	IIIa	adenocarcinoma	moderately-poorly
**GA0022**	M	≤5.0	T4a	N2	M0	IIIb	adenocarcinoma	moderately-poorly
**GA0023**	F	≤5.0	T4a	N2	M0	IIIb	adenocarcinoma	poorly
**GA0025**	F	≤5.0	T4a	N1	M0	IIIa	signet-ring cell carcinoma	poorly
**GA0033**	M	>5.0	T4a	N0	M0	IIb	adenocarcinoma	poorly
**GA0037**	M	≤5.0	T4a	N0	M0	IIb	adenocarcinoma	poorly
**GA0044**	M	lesion from liver	n/a	n/a	M1	IV	adenocarcinoma	moderately-poorly
**GA0046**	M	>5.0	T4a	N2	M0	IIIb	adenocarcinoma	poorly
**GA0055**	F	≤5.0	T4a	N3b	M0	IIIc	adenocarcinoma	moderately
**GA0060**	M	≤5.0	T4a	N3a	M0	IIIc	adenocarcinoma	moderately-poorly
**GA0075**	F	>5.0	T4a	N1	M0	IIIa	adenocarcinoma	moderately-poorly
**GA0080**	M	>5.0	T4a	N2	M0	IIIb	adenocarcinoma	moderately-poorly
**GA0098**	M	≤5.0	T4a	N0	M0	IIb	adenocarcinoma	moderately
**GA0114**	F	>5.0	T4a	N1	M0	IIIa	adenocarcinoma	moderately-poorly
**GA0119**	M	>5.0	T4a	N3b	M0	IIIc	adenocarcinoma	poorly
**GA0138**	M	>5.0	T2	N1	M0	IIa	adenocarcinoma	poorly
**GA0139**	M	>5.0	T4a	N0	M0	IIb	adenocarcinoma	well
**GA0151**	F	>5.0	T4a	N1	M0	IIIa	adenocarcinoma	moderately-poorly
**GA0152**	F	>5.0	T4a	N0	M0	IIb	adenocarcinoma	poorly
**GA2140**	M	>5.0	T4a	N0	M0	IIb	adenocarcinoma	moderately-poorly

* Stage of GC was classified according to 7th edition tumor-node-metastasis (TNM) classification recommended by the International Union Against Cancer.

Supplementary Table 2. Panel of primers used for mutation analyses.

Mutation	Primers
EGFR	
Exon 18	Forward 5′-CATGGTGAGGGCTGAGGTGA-3′<br>Reverse 5′-CCCCACCAGACCATGAGAGG-3′
Exon 19	Forward 5′-GTGCATCGCTGGTAACATCCA-3′<br>Reverse 5′-GGAGATGAGCAGGGTCTAGAGCA-3′
Exon 20	Forward 5′-CGCATTCATGCGTCTTCACC-3′<br>Reverse 5′-CTATCCCAGGAGCGCAGACC-3′
Exon 21	Forward 5′-TGGCATGAACATGACCCTGAA-3′<br>Reverse 5′-CAGCCTGGTCCCTGGTGTC-3′
KRAS	
Exon 2	Forward 5′-TTATGTGTGACATGTTCTAAT-3′<br>Reverse 5′-AGAATGGTCCTGCACCAGTAA-3′
Exon 3	Forward 5′-TCAAGTCCTTTGCCCATTTT-3′<br>Reverse 5′-TGCATGGCATTAGCAAAGAC-3′
Exon 4	Forward 5′-TTGTGGACAGGTTTTGAAAGA-3′<br>Reverse 5′-AGAAGCAATGCCCTCTCAAG-3′
BRAF	
Exon 15	Forward 5′-CTCTTCATAATGCTTGCTC-3′<br>Reverse 5′-GTGAATACTGGGAACTATG-3′
c-MET	
Exon 14	Forward 5′-TGGGCACTGGGTCAAAGTCTC-3′<br>Reverse 5′-AACAATGTCACAACCCACTGAGGTA-3′
Exon 16	Forward 5′-ATTAAATGTTACGCAGTGCTAAC-3′<br>Reverse 5′-GGTTGCAAACCACAAAAGTAT-3′
Exon 17	Forward 5′-GTATTCACTGTTCCATAATGAAGT-3′<br>Reverse 5′-GATGGCTGGCTTACAGCTAGTT-3′
Exon 18	Forward 5′-AACAGTAGATGCTTAGTTTATGCT-3′<br>Reverse 5′-AACAGATTCCTCCTTGTCACTT-3′
Exon 19	Forward 5′-TTCTATTTCAGCCACGGGTAAT-3′<br>Reverse 5′-ATGAAAGTAAAAGAGGAGAAACTC-3′
Exon 21	Forward 5′-CACCCTAAAGCCGAAATGCG-3′<br>Reverse 5′-CAAGGAGCAAAGAATATCGATGGC-3′
PI3KCA	
Exon 1	Forward 5′-CTCCACGACCATCATCAGG-3′<br>Reverse 5′-GATTACGAAGGTATTGGTTTAGACAG-3′
Exon 9	Forward 5′-GATTGGTTCTTTCCTGTCTCTG-3′<br>Reverse 5′-CCACAAATATCAATTTACAACCATTG-3′
Exon 20	Forward 5′-TGGGGTAAAGGGAATCAAAAG-3′<br>Reverse 5′-CCTATGCAATCGGTCTTTGC-3′
